# A Large Sporadic Intra-abdominal Desmoid-Type Fibromatosis in a Young Male: A Case Report

**DOI:** 10.3389/fsurg.2020.00060

**Published:** 2020-09-02

**Authors:** Natasha A. Sioda, Andre A. Wakim, Tina Wong, Shyamal Patel, Kathryn Coan, David Row

**Affiliations:** ^1^Creighton University School of Medicine, Phoenix, AZ, United States; ^2^Department of Surgery, Creighton University Arizona Health Education Alliance, Phoenix, AZ, United States; ^3^Department of Radiation Oncology, St. Joseph's Hospital and Medical Center, Phoenix, AZ, United States; ^4^Department of Surgery, St. Joseph's Hospital and Medical Center, Phoenix, AZ, United States

**Keywords:** retroperitoneal, intra-abdominal, desmoid, surgery, case report

## Abstract

Desmoid type fibromatosis (DTF) is a rare benign tumor of connective tissue origin. While these tumors are typically not malignant, they can exhibit aggressive growth patterns causing mass effect on surrounding organs. These tumors typically present in the extremities and abdominal wall, rarely occurring in the mesentery, and abdominal organs. Due to the rarity of this tumor and the variable size and origin, it is difficult to provide exact prognosis, recurrence, and treatment efficacy regarding desmoid tumors arising from the ileocolic mesentery. We present a case of a young male with a sporadic 31 × 25 × 12 cm DTF arising from the ileocolic mesentery that was complicated by mass effect on bowel and intra-abdominal organs requiring surgical intervention. On presentation, the patient exhibited weight gain and abdominal pressure. Abdominal distension without tenderness on palpation was noted on physical examination. The tumor biopsy confirmed the diagnosis of DTF. No evidence of familial adenomatous polyposis or Gardner syndrome was identified. The tumor was surgically excised and intimately associated with the bowel requiring ileocolonic resection with primary anastomosis. At 3-months follow up, surveillance MRI showed no residual or recurrent lesion. A multi-disciplinary approach to this patient's diagnosis and treatment allowed for an accurate diagnosis, efficient treatment, and follow up plan.

## Introduction

Desmoid-type fibromatosis (DTF) is a rare tumor that develops as a result of neoplastic proliferation of myofibroblasts within the musculo-aponeurotic stroma. They compose 0.03% of all tumors and <3% of soft tissue neoplasms (1). While they are non-metastasizing mesenchymal tumors, desmoid tumors may on rare occasion exhibit aggressive proliferative features that resulting in malignant complications secondary to mass effect. Particularly in the abdomen, they can cause intestinal obstruction or bowel ischemia (2). DTFs can be hereditary or occur sporadically, with the former being associated with familial adenomatous polyposis (FAP) or Gardner's syndrome. Patients with desmoid tumors carry an increased risk for FAP indicating the need for screening (3). The incidence of DTF in the general population is 2–4 per million, while the incidence among those with FAP is nearly 3% (4, 5). Among all reported DTFs, 5–15% occur in patients with FAP. These tumors most commonly affect young adults aged 25–35, and have a female-to-male preference of 2:1 (6). DTF can occur anywhere in the body, but they most commonly arise from the abdominal wall or extremities and rarely from the abdominal organs or mesentery (7). We present the workup and management of an unusual case of a 24-year-old male with a large sporadic 31 × 25 × 12 cm retroperitoneal desmoid tumor arising from the ileocolic mesentery requiring surgical intervention due to mass effect on abdominal organs.

## Case Description

A 24-year-old male was evaluated in the Emergency Department for abdominal discomfort. He has been transferred from a small rural hospital 200 miles away for a more extensive workup. The patient's primary concerns included a 4-weeks history of increasing abdominal pain with progressive weight gain. He denied nausea, vomiting, constipation, diarrhea, or any other associated symptoms. The patient was otherwise healthy with no prior surgical or medical interventions. His family history was significant only for a sister with thyroid cancer diagnosed at the age of 18. His social history was significant for previous service in the Marines. On physical examination, the patient was found to have a distended abdomen that was firm but non-tender on palpation with no guarding or rebound tenderness. Abdomen was non-tympanic on percussion.

## Diagnostic Assessment

**Figure 1:**
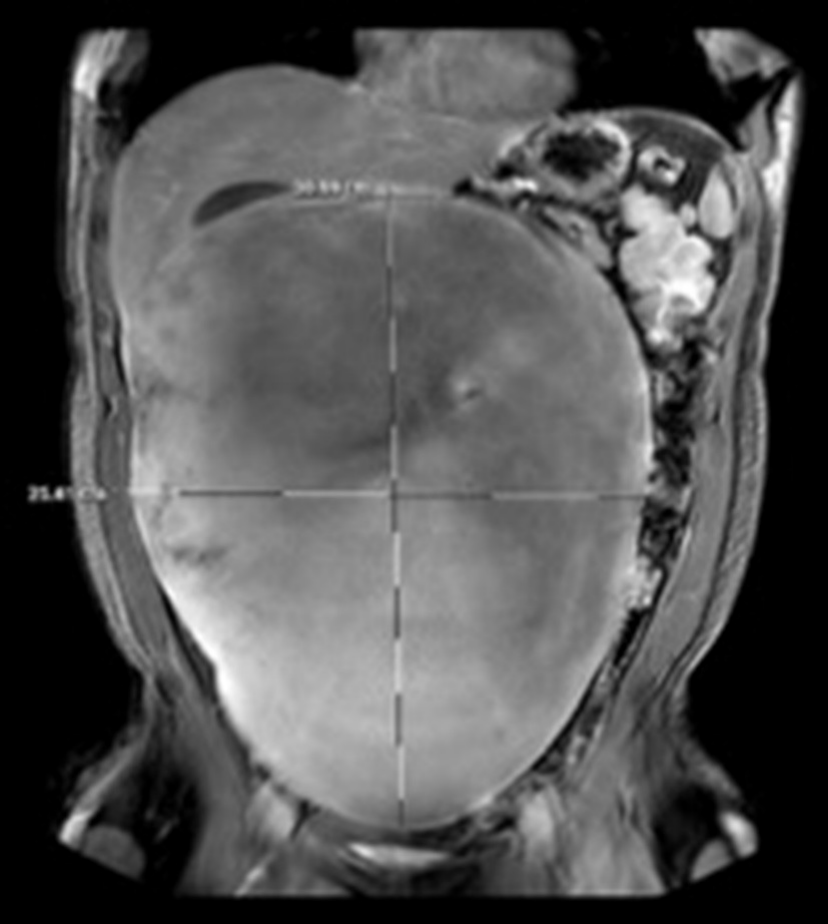
MR Abdomen and pelvis with contrast demonstrating 31 × 25 cm intra-peritoneal mass.

Computer tomography (CT) of the abdomen and pelvis demonstrated a large intra-abdominal mass measuring up to 30 cm. Surgery was consulted, and a magnetic resonance imaging (MRI) of the abdomen and pelvis was obtained to further delineate and characterize the mass. Imaging showed the presence of a 31 × 25 × 12 cm homogeneous intraperitoneal/mesenteric mass with internal vascularity that occupied the majority of the abdominal cavity with mass effect on the adjacent bowel loops and intrabdominal organs (Figure 1).

**Figure 2:**
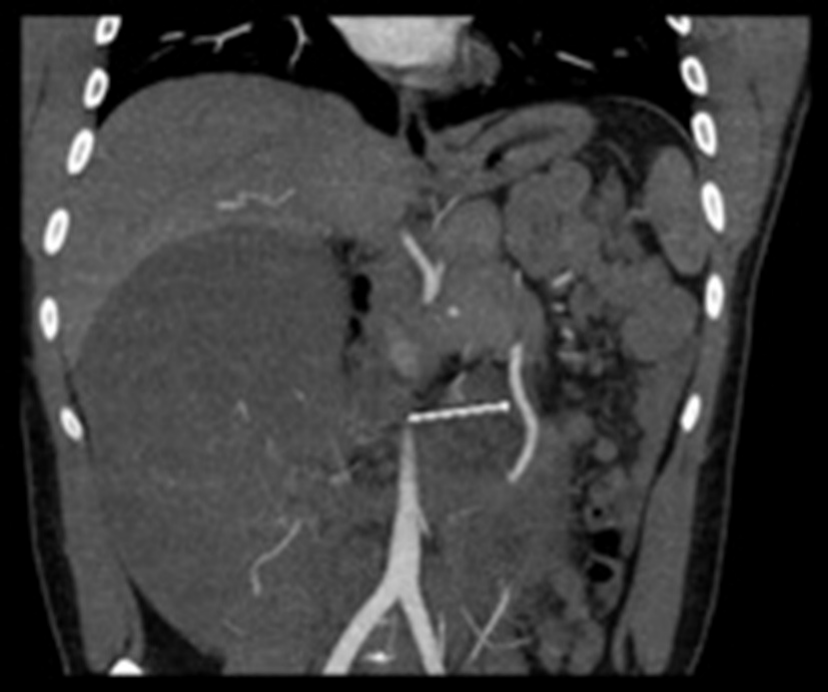
CT Angiogram arrow showing deviation of superior mesenteric artery to the left secondary to mass effect from large intra-abdominal mass. The 2nd branch of the SMA favored to represent the right colic branch courses anteriorly into and throughout the mass.

The initial differential diagnosis of this tumor included a large hematoma, GIST tumor, mesothelioma, or fibrosarcoma. An ultrasound-guided core needle biopsy of the mass was performed for histologic analysis. The neoplasm was negative for AE 1/3, D2-40, EMA, MUC4, and BCL2, weakly positive for CD68 and Beta-Catenin in the cytoplasm, and rarely positive for Calretinin in the nucleus. There was micro-focal positivity for muscle specific actin and scattered cells with cytoplasmic positivity for smooth muscle actin. Together with clinical presentation this was consistent with DTF. The case was discussed at our institutional multidisciplinary abdominal tumor board, and surgical resection was recommended as first line treatment. Neo-adjuvant radiation therapy was initially proposed to decrease tumor size. Surgical intervention was decided as the best treatment after imaging confirmed the tumor could be excised without neo-adjuvant radiation therapy. A CT angiogram was obtained for preoperative planning purposes in order to better visualize the relationship between the tumor and the mesenteric vessels. The vascular pedicle for the tumor was noted to arise from a distal branch of the SMA near the ileocolic region while sparing the remainder of the surrounding vasculature (Figure 2). Additional details regarding the patient's delivery of care is outlined in Table 1. The only diagnostic challenge encountered was the patient's commute to the hospital where he received treatment.

**Table 1:** Timeline of patient care.

**Date**	**Event**
Early June, 2019	Blunt abdominal trauma from bicycle handlebars
July 2, 2019	Presented to ED with abdominal distension Abdominal mass found on physical exam CT of abdomen demonstrated 30cm mass Admitted by general surgery
July 4, 2019	MRI of abdomen and pelvis w/wo contrast showed visceral involvement
July 5, 2019	US-guided biopsy of mass performed under sedation Discharged from hospital
July 9, 2019	Case was discussed at tumor board Surgery with radiation therapy was decided
July 11, 2019	Surgical oncology office visit, radiation oncology office visit
July 16, 2019	Pathology report revealed desmoid-type fibromatosis
July 18, 2019	Pre-operative consult with surgical oncology
July 19, 2019	CT of abdomen with IV contrast demonstrated mass could be resected without neoadjuvant radiation therapy
July 23, 2019	Mass was surgically excised
July 27, 2019	Patient was discharged from hospital
July 30, 2019	Pathology report confirmed desmoid-type fibromatosis with R0 margins
August 1, 2019	Surgical oncology office visit confirmed appropriate recovery
November 6, 2019	MRI of abdomen and pelvis showed no evidence of recurrence

## Therapeutic Intervention

After appropriate counseling, the patient agreed to proceed with open surgical tumor resection. Neoadjuvant radiation therapy was considered due to initial diagnosis of possible fibrosarcoma, but after confirming diagnosis of desmoid tumor and reviewing imaging results, surgery without radiation was determined to be the best course of action. Upon making the laparotomy incision, the tumor was readily appreciable and appeared well-defined and encapsulated. Initial attempts to deliver the tumor out of the abdomen was met with difficulty due to dense adhesions between the tumor and the right lateral abdominal wall, liver, and right retroperitoneum. These adhesions were carefully released. Posteriorly, the tumor was found to be intimately associated with the terminal ileum, cecum, and ascending colon with loss of tissue planes. The vascular pedicle was palpated and confirmed to originate from the ileocolic artery, as indicated by preoperative imaging. The decision was made to perform an en bloc resection of the tumor along with the terminal ileum, cecum, and ascending colon with primary ileocolic anastomosis. The patient tolerated the surgery well, ambulating beginning on post-operative day 1 and passing flatulence on post-operative day 3. By post-operative day 4, he was tolerating a full diet and had a bowel movement, so he was discharged home.

## Follow-Up and Outcomes

**Figure 3:**
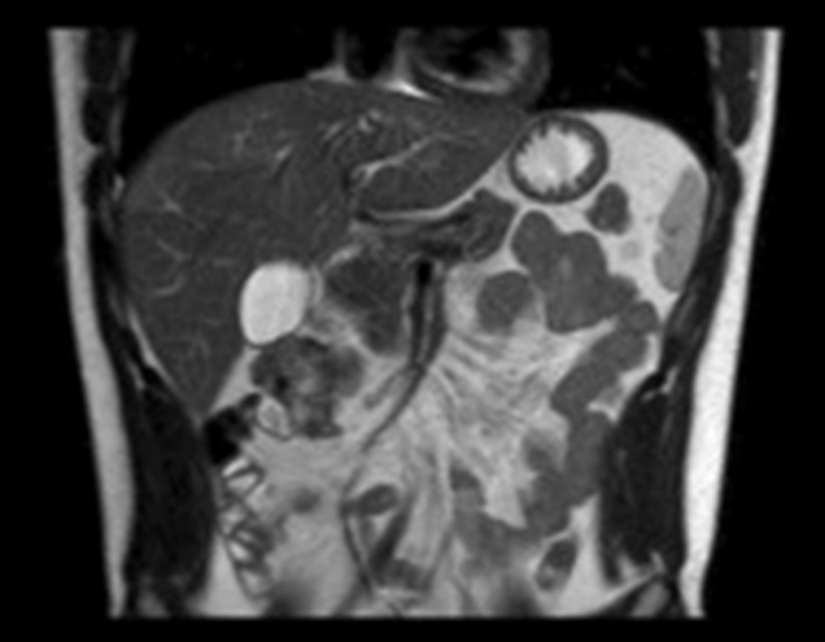
Coronal MR abdomen and pelvis at 3 month follow-up showing no recurrence.

Final pathology of the surgical specimen revealed a desmoid tumor with involvement of the muscularis of the small bowel and colon. Surgical margins on the small bowel and colon were negative for tumor. There was extension of tumor peripherally to the peritoneal surface. Genetic testing was performed and demonstrated no mutation of the APC gene; and given that and the patient was not experiencing any gastrointestinal symptoms, a colonoscopy was deferred at this time. At his post-operative visit 9 days out from surgery, the patient was doing well and had returned to his baseline physical activity. He denied abdominal pain, difficulties with bowel movements, or any other symptoms. His 3-months surveillance MRI showed no residual or recurrent lesion (Figure 3). The patient continues to adhere to surveillance with clinical evaluation and repeat MRI every 3–6 months at an outside institution.

## Discussion

DTF is an uncommon stromal tumor that can develop sporadically or in association with hereditary syndromes such as FAP or Gardner Syndrome. While benign in nature, these tumors can become symptomatic from its size alone and warrant surgical intervention. In this case report, we describe 24-year-old male with a large sporadic retroperitoneal desmoid tumor arising from the ileocolic mesentery.

The rare occurrence and variable presentations of DTF have limited our ability to thoroughly understand and investigate its prognosis, recurrence, and treatment efficacy. This is important to consider since recurrence may contribute to the overall long-term survival of the patient. One retrospective analysis showed that the median overall survival following desmoid tumor diagnosis was 10.2 years while in those that experienced recurrence, this was reduced to 6.9 years (8). In another retrospective study of 203 extra-abdominal desmoid tumors, patients presenting with primary disease experienced a better 10-years disease free survival rate than those with recurrent disease (76 vs. 59% in 10 years) (9). With regards to intra-abdominal tumors, each subsequent recurrence had an increased risk of compression and intimate association with vital organs and vessels limiting the resectability of the tumor. Considering our patient in particular, his tumor occupied the majority of the abdominal cavity leading to mass effect on the abdominal organs and bowel. The severe morbidity associated with recurrence warrants continuous monitoring with an MRI every 3 months in our patient.

Current guidelines favor conservative management of DTF with observation/surveillance, as growth of desmoid tumors have been shown to be slow growing or halt spontaneously in up to 50% of tumors (10–14). The size of tumors in recent articles supporting conservative management of DTF were predominantly 6 cm in size and the progression free survival rate of tumors >10 cm was unable to be determined due to insufficient data (11–15). Moreover, the majority of cases documenting spontaneous regression with conservative management were extra-abdominal (11–18). In 2015, there was a proposed consensus treatment algorithm determined by the European Organization for Research and Treatment of Cancer (EORTC), Soft Tissue and Bone Sarcoma Group (STBSG), and Sarcoma Patients EuroNet (SPAEN) (17). The proposed treatment algorithm acknowledged that while there has been spontaneous regression observed at all anatomical sites of desmoid tumors, surgery remains the main treatment for progressive and operable intra-abdominal tumors (17). Gronchi also detailed a step wise treatment plan for sporadic DTF approved by the French and Italian Sarcoma group. While supporting an initial waiting period, they also acknowledged the need for immediate medical or surgical intervention given location (head, neck, pelvis, or intrabdominal cavity), size, and symptoms exhibited by patient (16). Our patient had an unusually large symptomatic tumor with documented mass effect on intra-abdominal organs and bowel in addition to worsening abdominal symptoms over a short time frame of 4 weeks. This indicated an aggressive growth pattern and need for surgical intervention rather than conservative management. Given the intra-abdominal location and size (31 × 25 × 12 cm) of the DTF, further risk of vessel compression as well as worsening mass effect on bowel and intra-abdominal organs indicated surgical intervention. Recently there was a documented case of spontaneous regression in a young asymptomatic patient with a 10.4 × 6.6 cm inoperable intra-abdominal desmoid tumor. This case details the possible role for conservative management in asymptomatic patients with inoperable intra-abdominal desmoid tumors (19). However, our patient was symptomatic with a DTF of greater magnitude and mass effect on abdominal organs requiring surgery. Considering our patient's presentation, a multidisciplinary approach with detailed preoperative imaging and histologic analysis were performed to assist with surgical planning.

There is controversy surrounding the role of R0 resection in preventing recurrence (20). Larger retrospective studies have shown higher recurrence rates for patients with R1 margins compared to R0 margins while other studies have shown that margin status is a predictor of recurrence with univariate analysis but not on multivariate analysis (6). Ultimately, Howard et al. reports that although R0 resection is preferred, recurrence rates following R0 and R1 resection may be similar, particularly when surgery is complemented with adjuvant therapy. An example of adjuvant therapy is the tyrosine kinase inhibitor sorafenib. A recent randomized control trial demonstrated that patients with advanced (inoperable or symptomatic) DTFs that are treated with sorafenib have an improved 2-years progression-free survival rate compared to the placebo group (81 vs. 36%) (21).

DTFs have a strong association with FAP; while FAP only occurs in one in 8,300–37,600 births, 5–15% of all DTFs occur in individuals with FAP (22, 23). FAP is the result of a germline mutation in the adenomatous polyposis coli (APC) gene, a tumor suppressor gene that normally regulates the β-catenin/wnt growth factor pathway. Germline mutations in APC genes have been found in both sporadic DTFs and those associated with FAP (23). B-catenin is yet another gene that is often involved in DTF. It is a proto-oncogene normally found in the cytoplasm, whose activation leads to nuclear translocation and transcription factors activation (24). B-catenin has been found to be mutated in up to 71–91% of sporadic cases of DTF (6). Our patient tested negative for mutations in the APC gene and had weak cytoplasmic positivity with no nuclear positivity for β-catenin, suggesting that neither APC nor β-catenin played a role in the pathogenesis of his tumor.

Interestingly, up to 30% of sporadic DTF cases have been demonstrated to be preceded by trauma (25) including previous motor vehicle accidents (26) and cesarean section (27). It has been proposed that this association may in fact be the result of aberrant proliferation after a traumatic insult, with an underlying over-activation of the molecular signaling pathway for normal wound healing (4). Given that our patient served in the Marines, the cause of his DTF may have been due to unidentified antecedent abdominal trauma. He also reported bicycle trauma to his abdomen 1 month prior to presentation of symptoms.

Our patient was medically compliant throughout his treatment and follow up allowing for sufficient and optimal care. He had a stable recovery post-operatively and adequate hospital resources were provided to him. Other than an extended commute to the medical center, the patient did not have other diagnostic challenges limiting clinical management or subsequent follow up compliance. This allowed for multiple multi-disciplinary discussions, efficient operative planning, and continued monitoring. As a result of how recent this patient received treatment, this case report does not provide adequate insight into recurrence or long term prognosis of this type of neoplasm. Reports and studies involving long term follow-up and patient outcomes will be valuable in deepening the understanding of DTF.

## Conclusion

DTF is a rare, benign tumor of myofibroblast origin. While they most commonly arise in the extremities or abdominal wall we present a rare occurrence in the ileocolic mesentery. Long term recurrence in mesenteric desmoid tumors remains undetermined given its rarity and variable presentation. Recent guidelines recommend more conservative approaches and watchful observation, but our case emphasized the need for immediate surgical intervention considering location and size. Our case emphasized the importance of a multidisciplinary approach in establishing an accurate diagnosis and in providing the proper treatment with the necessary perioperative considerations. This case presented a rare presentation of ileocolic mesentery desmoid tumor and demonstrated the complexities in managing patients with large intra-abdominal desmoid tumors.

## Patient Perspective

I managed to remain positive throughout the process mostly because I felt that I needed to be strong for my wife. I experienced anxiety for nearly 3 weeks because an accurate diagnosis did not appear until 3 weeks after initial discovery. The rarity of my tumor caused many doctors to visit my room at once which initially was not a problem until my wife and I would be asked questions about a diagnosis we hadn't gotten yet. Although the staff at the hospital were kind and caring, I felt completely in the dark about my situation. I first arrived with the thought of emergency surgery but for 4 days my wife and I were never given a straight answer. Attendings, residents, interns would shuffle into the room. I didn't mind the attentiveness but the question about surgery was never answered. Instead we were asked about cancer centers without ever being told it was cancer. After several days I received a biopsy and MRI only to be told that the general surgery team would not operate due to the foreknown size of the tumor. I was quickly referred to a nearby cancer center. Our surgeon and nurse were a breath of fresh air. The surgeon was clear, concise, and informative. During every visit he answered all of our asked and unasked questions. He would provide us with hope and reassurance and his confidence was contagious. I feel that the decision of treatment was beyond us. We simply wanted the best outcome no matter what and our surgeon, with all his confidence and experience, gave us just that. From initial discovery to removal of my tumor was 3 weeks. Things moved too quickly for us to have an opinion for my treatment. My surgeon gave us the confidence to leave the treatment decisions to the experts. I was one of the lucky ones. I did not have metastatic cancer. Surgery removed the entirety of my tumor with negative margins. My genetic testing was negative and all follow-up MRIs have been negative as well. I want to extend a thank you to all who treated me with kindness. I will strive to be just as kind, attentive, and caring as those who helped me when I most needed it.

This condition taught me true pain, fear, and anxiety. It reminded me that life can change at a blistering pace. This tumor also reminded me the importance of exercising and maintaining a proper diet. During hot summer days in my hometown I am thankful for the dry air that fills my lungs and the burn I feel throughout my body because I can run, because I am strong. After the removal of my tumor life has somehow returned to normalcy but it does serve as a reminder to be grateful and fierce. This is my second chance. This condition has reminded me to pursue what I value in life. I've chosen to follow a career in healthcare despite years of aviation experience in the Marine Corps. I thank God and the staff at each hospital I received care for everything they have done for me. I've grown a new appreciation for those who choose to serve our fellow human beings and hope that 1 day I can have the honor of working alongside them.

## Data Availability Statement

The original contributions presented in the study are included in the article/Supplementary Material, further inquiries can be directed to the corresponding author/s.

## Ethics Statement

Written informed consent was obtained from the individual for the publication of any potentially identifiable images or data included in this article.

## Author Contributions

NS and AW contributed to the design, revision, format, acquisition of the patient's case presentation, introduction, discussion, and informed consent of the patient. TW, KC, DR, and SP contributed to extensive draft and revision of the case report and providing feedback and suggestions. All authors contributed to the article and approved the submitted version.

## Conflict of Interest

The authors declare that the research was conducted in the absence of any commercial or financial relationships that could be construed as a potential conflict of interest.
