# Supplementary figures and images for: A Large Sporadic Intra-abdominal Desmoid-Type Fibromatosis in a Young Male: A Case Report
Source: Front Surg. 2020 Sep 2;7:60. doi: 10.3389/fsurg.2020.00060 (PMC7493805; doi:10.3389/fsurg.2020.00060)

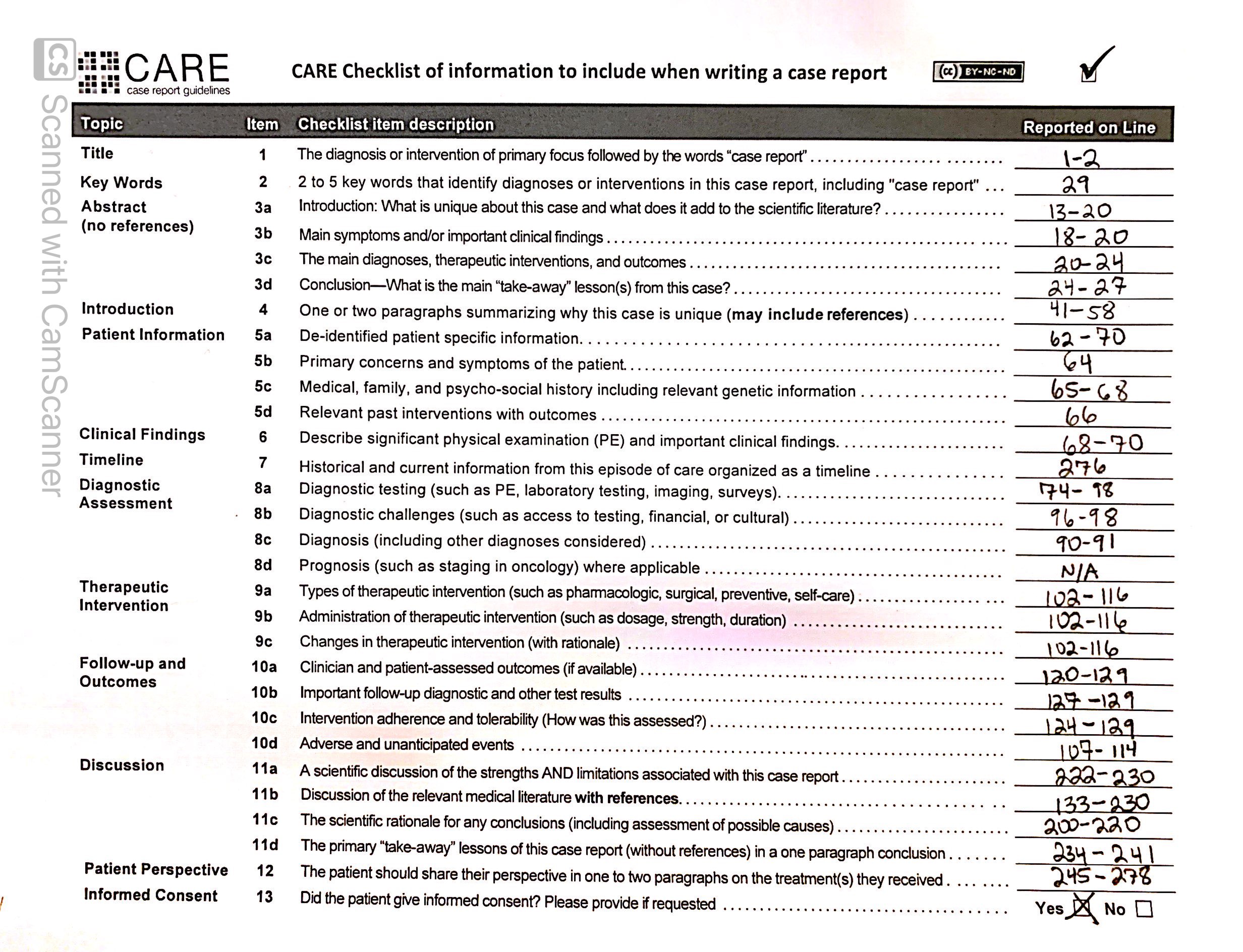

Supplement: Supplementary file 1 [file Image_1.JPEG]
